# TDP-43 pathology in the retina of patients with frontotemporal lobar degeneration

**DOI:** 10.1007/s00401-023-02623-8

**Published:** 2023-08-19

**Authors:** Anke A. Dijkstra, Tjado H. J. Morrema, Frederique J. Hart de Ruyter, Priya Gami-Patel, Frank D. Verbraak, Johannes F. de Boer, Femke H. Bouwman, Yolande A. L. Pijnenburg, Jurre den Haan, Annemieke J. Rozemuller, Jeroen J. M. Hoozemans

**Affiliations:** 1 Department of Pathology, Amsterdam UMC, Location VUmc, Amsterdam Neuroscience, Amsterdam, The Netherlands; 2 Swammerdam Institute for Life Sciences, University of Amsterdam, Amsterdam, The Netherlands; 3 Amsterdam UMC, Location VUmc, Alzheimer Center, Neurology, Amsterdam, The Netherlands; 4 Ophthalmology Department, Amsterdam UMC, Location VUmc, Amsterdam, The Netherlands; 5 Department of Physics, Bio Laser Lab, VU University, Amsterdam, The Netherlands

Frontotemporal dementia (FTD) is a clinically heterogeneous disease, characterized by behavioural, language and movement symptoms. The underlying pathology is termed frontotemporal lobar degeneration (FTLD) and consists of distinct underlying pathologies including aggregation of transactive response DNA-binding protein 43 (TDP-43), tau and fused-in-sarcoma (FUS) [7]. Approximately half of the FTLD cases have underlying TDP-43 pathology (FTLD-TDP), which can either be caused by a genetic mutation, with the most frequent being a hexanucleotide repeat expansion in *C9orf72* or a mutation in progranulin (*PRGN*) or have a sporadic cause with no family history of dementia [7]. Currently, no ante-mortem biomarkers are available to distinguish between the different pathological subtypes, leaving challenges for diagnosis and therapeutic interventions. There is a growing interest in the eye, and in particular the retina, as a potential imaging biomarker for neurodegenerative diseases. As an extension of the brain, the retina displays anatomical and functional similarities. The accessibility of the retina provides a ‘window’ into the brain and a potential for noninvasive imaging of pathological changes in patients with a neurodegenerative disease. Previous research in human studies showed amyloid β (Aβ), phosphorylated tau and α-synuclein deposits in Alzheimer’s disease (AD), progressive supranuclear palsy (PSP) and Parkinson’s disease (PD) post-mortem retinas [5, 10, 12]. Recently, increased signals for phosphorylated TDP-43 (pTDP-43) were observed in the retina of cases with amyotrophic lateral sclerosis (ALS) [9]. Earlier it was reported that in a donor with ALS carrying a *C9orf72* mutation, dipeptide pathology was present in the retina in the absence of TDP-43 pathology [2].

There are limited data on retinal changes in FTLD. In donors with FTLD due to *PRGN* mutation, retinal changes have been observed and include thinning and formation of fluorescent lesions, which precede symptoms of FTD [13, 14]. In a *PRGN* mouse model, neurodegeneration and nuclear depletion of TDP-43 was reported without TDP-43 aggregation [14]. Here we investigated the mislocalization of TDP-43 and presence of pTDP-43 in FTLD-TDP retina.

Post-mortem retina tissue was obtained from 7 donors with FTLD-TDP, of which four had a *C9orf72* repeat expansion, one *PRGN* mutation and two were genetically sporadic (Netherlands Brain Bank, Amsterdam, The Netherlands). Pathologically, donors presented with FTLD-TDP types A (*PRGN*), B (*C9orf72*), C (sporadic) and E (sporadic) [6]. We also included donors with FTLD–tau (*n* = 5), FTLD-FUS (*n* = 1), AD with limbic-predominant age-related TDP-43 depositions (LATE) (*n* = 2) and without (*n* = 1), donors with ALS due to TDP-43 (ALS-TDP (*n* = 2)) and neurologically healthy controls (*n* = 6). Retinal TDP-43 pathological burden in FTLD-TDP donors was scored into none, few, frequent and abundant inclusions. Details and pTDP-43 scores of the donors are listed in Supplementary File 1. Immunohistochemical stainings were performed for (pan)TDP-43, phosphorylated TDP-43 (pTDP-43), p62 (SQSTM1), and the dipeptide repeat proteins (polyGA and polyGP). Double-fluorescence with calbindin D28K and calretinin was performed to identify horizontal cells and amacrine cells, respectively (for detailed procedures see Supplementary File 1).

In *C9orf72* and *PRGN* mutation carriers, as well as the sporadic FTLD-TDP donors, TDP-43 aggregates were observed in the outer plexiform layer of the retina. No colocalization was found with markers for horizontal or amacrine cells. No TDP-43 inclusions were observed in the retina of FTLD-tau, ALS, AD, and neurologically healthy control donors. Interestingly, all *C9orf72* mutation carriers showed abundant presence of p62 and display aggregation of dipeptides linked to the hexanucleotide repeat expansion in the inner nuclear layer of the retina [8]. The FTLD-TDP donors with advanced cortical and subcortical pathology, also showed the most abundant TDP-43 pathology (Fig. 1). One sporadic donor presented with a fast disease progression and had inclusions characteristic for FTLD-TDP type E. Characteristic for FTLD-TDP type E are small granular TDP-43 inclusions that are both P62- and ubiquitin negative [6]. In the retina, only few TDP-43 granular inclusions were observed, which is in concordance with the TDP-43 pattern observed in the brain.

**Fig. 1:**
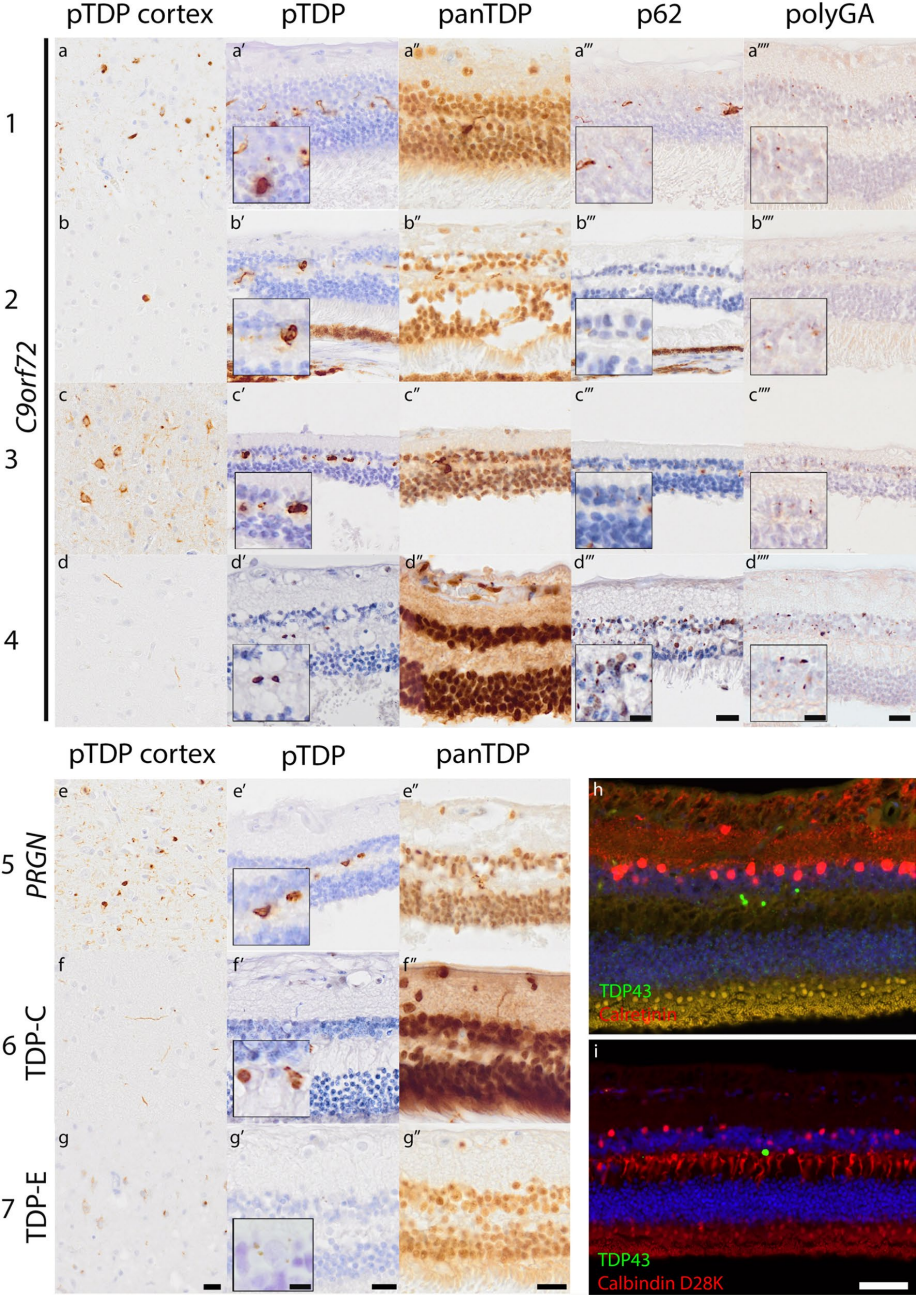
Pathological features of FTLD-TDP are present in the retina. **a**–**g** pTDP-43 immunostaining in the frontal cortex shows the typical distribution specific for FTLD-TDP subtypes. **a**′–**g**′ pTDP-43 immunoreactive aggregates are observed in the outer plexiform layer (OPL) in FTLD-TDP cases. **a**″–**g**″ Aggregates in the OPL are observed with panTDP immunostaining. **a**′′′–**d**′′′ In C9orf72 hexanucleotide repeat expansion carriers P62 immunostaining shows larger aggregates in the OPL, and small aggregates in the inner nuclear layer (INL). **a**″″–**d**″″ PolyGA immunoreactive aggregates are observed in the INL in C9orf72 hexanucleotide repeat expansion carriers. Scale bar: 20 µm (applicable to all images), scale bar inserts: 10 µm (applicable to all inserts). Immunostaining shown with DAB (brown) and nuclei are counterstained with hematoxylin (blue). **h**, **i** Fluorescent double-immunostainings for pTDP-43 (green) with calbindin D28K and calretinin. h shows presence of calretinin (red) in amacrine cells in the INL and its processes in the IPL. **i** shows detection of calbindin D28K (red) in cones, horizontal cells and bipolar cells. Scale bar: 50 µm (applicable to both images). Nuclear staining with DAPI (blue)

The ALS patients included in the current study did not carry the *C9orf72* hexanucleotide repeat expansion and showed no retinal or cortical TDP-43/dipeptide pathology. Recently, an increased signal for pTDP-43 in the retina was reported in 6 ALS donors [9]. This contradicts with an earlier report and the results from 2 donors in the current study [2]. While there is increasing evidence for in vivo retinal damage using ophthalmologic examination and post-mortem signs of neurodegeneration in cases with ALS, additional studies are needed to address the presence of pTDP-43 in retina in the context of ALS [11]. We observed pTDP-43 aggregates in the retina of *C9orf72* mutation carriers. This suggests that genetic factors play a role in the occurrence of TDP-43 aggregates. On the other hand, we also observed TDP-43 aggregation in sporadic FTLD-TDP cases. Interestingly, TDP-43 inclusions in the retinal internuclear layer have been reported in subjects with chronic traumatic encephalopathy (CTE) [3]. This supports that TDP-43 inclusions in the retina can occur independent from underlying mutations. The absence of pTDP-43 positive aggregates in AD with limbic TDP-43 depositions suggests that the occurrence of TDP-43 aggregates in the retina relates to the severity of TDP-43 pathology in the brain, since within these limbic cases the TDP-43 pathology remains localized. In addition, timing could play a role as TDP-43 aggregation is secondary to AD-related pathology and mostly occurs in a later stage in AD [1, 4]. Additional studies are needed to determine if retinal TDP-43 aggregates contribute to a progressive cortical pathology or if they should be regarded as a primary retinal pathology.

To summarize, we report that manifestations of pathological TDP-43 are present in the retina of donors with FTLD-TDP. In donors with *C9orf72* mutations, dipeptide pathology is also present in the retina. In this study, we observed no TDP-43 inclusions in the retina of 2 ALS donors without cortical TDP-43 depositions or AD patients with limbic TDP-43 pathology, suggesting that retinal TDP-43 pathology reflects the cortical involvement of TDP-43 aggregation. These findings provide opportunities for retinal TDP-43 aggregation as a biomarker for FTLD-TDP, using non-invasive retinal imaging techniques with the purpose of diagnosing and monitoring progression of FTLD-TDP.

### Supplementary Information

Below is the link to the electronic supplementary material.

Supplementary file1 (DOCX 25 KB)

## Data Availability

Most data generated or analyzed during this study are included in this published manuscript and the supplementary material. Additional data are available from the corresponding author upon reasonable request.
