# Supplementary material for: TDP-43 pathology in the retina of patients with frontotemporal lobar degeneration
Source: Acta Neuropathol. 2023 Aug 19;146(5):767–70. doi: 10.1007/s00401-023-02623-8 (PMC10564657; doi:10.1007/s00401-023-02623-8)
Supplement: Supplementary file 1 — Supplementary file1 (DOCX 25 KB) [file 401_2023_2623_MOESM1_ESM.docx]

**Supplemental file**

**Materials and Methods**

**Brain and retina post mortem tissue**

Post-mortem eyes and brain tissue was collected prospectively from 2009 until 2022 by the Netherlands Brain Bank (Amsterdam, The Netherlands, https://www.brainbank.nl). The donor program of the Netherlands Brain Bank was approved by the VUmc medical ethics committee. All donors consented with the usage of their tissue and clinical records for research purposes in compliance with ethical standards. Brain autopsies were performed according to the Code of Conduct of Brain Net Europe [5].

Brain autopsy was performed within 12 hours post-mortem after which brain tissue was formalin-fixed (10%; 4 weeks) and embedded in paraffin. Neuropathological diagnosis was performed according to the guidelines of the BrainNet Europe Consortium including assessment of Braak-stage for Lewy bodies (Braak LB stage) [1] and Alzheimer’s disease neuropathological changes [4, 7] including Thal-phase for Aβ [8], Braak-stage for NFTs [2] and CERAD score for neuritic plaque pathology [6]. Retinal tissue preparation was performed as described before [3]. Briefly, the cornea and lens of the eye was removed, and filled with tissue-tek O.C.T. compound (Sakura, Tokyo, Japan). Either the eye was frozen using iso-pentane at −90 °C and stored at −80 °C, or the eye was directly collected in 4% paraformaldehyde (PFA) and stored for 48 h before further processing. Frozen eyes were defrosted at room temperature (RT) in PFA for 48 h prior to dissection. The eye was dissected through the horizontal and vertical axis, resulting in temporal-superior, temporal-inferior, nasal-superior and nasal-inferior quadrants [3].

**Immunohistochemistry**

Paraffin embedded formalin‐fixed tissue sections (10 μm thick) were cut from the retina as described previously [3]. After deparaffinization, the sections were washed three times for 5 minutes phosphate buffer saline (PBS; pH 7.4), and after washing endogenous peroxidase activity was blocked with 0.3% H_2_O_2_ in PBS for 30 min. Sections were then treated in 0.1 M citrate buffer (pH 6.0) heated by autoclave (at 121°C for 10 min) for antigen retrieval. Tissue sections were incubated one hour or overnight at room temperature with primary antibodies (Table 1) diluted in antibody diluent (Sigma-Aldrich, Saint Louis MO, USA). After incubation, sections were washed with PBS and incubated with HRP‐labeled Envision (DAKO, Glostrup, Denmark) for 30 min. Color was developed with 3,3′‐diaminobenzidine (DAB; DAKO). Sections were counterstained with haematoxylin and mounted using Quick D (Klinipath; Duiven, the Netherlands).

**Double immunofluorescensce**

Colocalization of pTDP43 with retinal neuronal cells was visualized using antibodies against calbindin D28K and calretinin. Deparaffinization, citrate antigen retrieval using an autoclave and blocking of endogenous peroxidase activity were performed as described above. Sections were incubated overnight at RT with a mix of anti-calbindin D28K + pTDP43 or anti-calretinin + pTDP43 diluted in antibody diluent (Sigma-Aldrich). The pTDP43 was visualized using a 30 minute incubation of anti-mouse Envision (K4000; Dako), followed by incubation of 10 minutes with TSA Plus Fluorescein 488 (Akoya Biosciences, USA), and calbindin D28K and calretinin with goat anti-rabbit IgG Alexa Fluor Plus 594 (Thermo Fisher Scientific, Waltham MA, USA) as a secondary step in a 1:250 dilution in antibody diluent for 1 h. In between incubations, sections were washed with PBS at RT. After completion of the stainings sections were coverslipped with DAPI Fluoromount-G (Southern Biotech, Birmingham AL, USA). Omission of primary antibody and staining of single antibodies were used as technical controls.

**Table 1.** Antibodies and conditions used in this study

| **Antibody** | **Manufacturer** | **Product number** | **AR** | **Dilution** | **Incubation time** |
| --- | --- | --- | --- | --- | --- |
| panTDP43 | Abnova GmbH, Taipei, Taiwan | H00023435-M01 | CB | 1:8000 | overnight |
| pTDP43 pSer409/410 | Cosmo Bio, Tokio, Japan | clone 11‐9 | CB | 1:4000 | 1 hour |
| p62 lck ligand | BD Biosciences, San Jose CA, USA | clone 3/P62 | CB | 1:1000 | 1 hour |
| Anti C9orf72 polyGA | Cosmo Bio, Tokio, Japan | C9GA1312 | CB | 1:2000 | overnight |
| Anti C9orf72 polyGP | Cosmo Bio, Tokio, Japan | C9GP1312 | CB | 1:2000 | overnight |
| Calbindin D28K | Atlas Antibodies, Stockholm, Sweden | HPA023099 | CB | 1:1000 | overnight |
| Calretinin | Thermo Fisher Scientific, Waltham MA, USA | pa5-16681 | CB | 1:400 | overnight |

Abbreviations: AR, antigen retrieval method; CB, citrate buffer heat pretreatment

**Table 2.** Cases used in this study

|  |  |  |  |  | **pTDP-43 score** | | | |
| --- | --- | --- | --- | --- | --- | --- | --- | --- |
| **Case #** | **Neuropathology diagnosis** | **Age** | **Sex** | **ABC score** | **NS** | **NI** | **TI** | **TS** |
| 1 | FTD-TDP-B (*C9orf72*) | 74 | F | n.a. | 3 | 3 | 3 | 3 |
| 2 | FTD-TDP-B (*C9orf72*) | 75 | M | 2,2,1 | 1 | 0 | 1 | 0 |
| 3 | FTD-TDP-B (*C9orf72*) | 65 | M | 1,1,0 | 3 | 3 | 3 | 3 |
| 4 | FTD-TDP-B (*C9orf72*) | 70 | M | n.a. | 1 | 1 | 1 | 3 |
| 5 | FTD-TDP-A (*PRGN*:S82valfsX174)) | 58 | M | 0,0,0 | 1 | 1 | 3 | 2 |
| 6 | FTLD-TDP-C (sporadic) | 72 | M | n.a. | 2 | 3 | 1 | 1 |
| 7 | FTD-TDP-E (sporadic) | 51 | F | n.a. | 1 | 0 | 1 | 0 |
| 8 | FTD-FUS (aFTLDU) | 50 | M | 0,0,0 | 0 |  |  |  |
| 9 | FTD-TAU-MAPT (A296A) | 65 | M | n.a. | 0 |  |  |  |
| 10 | CBD | 59 | M | 2,1,0 | 0 |  |  |  |
| 11 | CBD | 74 | F | 2,1,0 | 0 |  |  |  |
| 12 | PSP | 65 | F | 1,1,1 | 0 |  |  |  |
| 13 | PSP | 65 | M | 1,1,0 | 0 |  |  |  |
| 14 | ALS-TDP | 68 | M | n.a. | 0 |  |  |  |
| 15 | ALS-TDP | 71 | M | 1,1,0 | 0 |  |  |  |
| 16 | AD; no TDP | 61 | F | 3,3,3 | 0 |  |  |  |
| 17 | AD; limbic TDP | 82 | M | 3,3,3 | 0 |  |  |  |
| 18 | AD; limbic TDP | 80 | F | 3,2,2 | 0 |  |  |  |
| 19 | Control | 49 | M | 0,0,0 | 0 |  |  |  |
| 20 | Control | 76 | F | 0,1,0 | 0 |  |  |  |
| 21 | Control | 57 | F | 0,0,0 | 0 |  |  |  |
| 22 | Control/depression | 68 | M | 0,0,0 | 0 |  |  |  |
| 23 | Control | 80 | M | 0,1,0 | 0 |  |  |  |
| 24 | Control | 63 | M | n.a. | 0 |  |  |  |

Abbreviations: NS, nasal superior; NI, nasal inferior; TI, temporal inferior; TS, temporal superior**;** n.a. , not available; F, female; M, male.

ABC score for amyloid beta plaques, neurofibrillary tangles and neuritic plaques according to Hyman et al. [4]. pTPD-43 score for the retina: 1) none, 2) few, 3) frequent, 4) abundant.

**References**

1 Alafuzoff I, Ince PG, Arzberger T, Al-Sarraj S, Bell J, Bodi I, Bogdanovic N, Bugiani O, Ferrer I, Gelpi Eet al (2009) Staging/typing of Lewy body related alpha-synuclein pathology: a study of the BrainNet Europe Consortium. Acta Neuropathol 117: 635-652 Doi 10.1007/s00401-009-0523-2

2 Braak H, Braak E (1991) Neuropathological stageing of Alzheimer-related changes. Acta Neuropathol 82: 239-259 Doi 10.1007/BF00308809

3 Hart de Ruyter FJ, Morrema THJ, den Haan J, Netherlands Brain B, Twisk JWR, de Boer JF, Scheltens P, Boon BDC, Thal DR, Rozemuller AJet al (2023) Phosphorylated tau in the retina correlates with tau pathology in the brain in Alzheimer's disease and primary tauopathies. Acta Neuropathol 145: 197-218 Doi 10.1007/s00401-022-02525-1

4 Hyman BT, Phelps CH, Beach TG, Bigio EH, Cairns NJ, Carrillo MC, Dickson DW, Duyckaerts C, Frosch MP, Masliah Eet al (2012) National Institute on Aging-Alzheimer's Association guidelines for the neuropathologic assessment of Alzheimer's disease. Alzheimers Dement 8: 1-13 Doi 10.1016/j.jalz.2011.10.007

5 Klioueva NM, Rademaker MC, Dexter DT, Al-Sarraj S, Seilhean D, Streichenberger N, Schmitz P, Bell JE, Ironside JW, Arzberger Tet al (2015) BrainNet Europe's Code of Conduct for brain banking. J Neural Transm (Vienna) 122: 937-940 Doi 10.1007/s00702-014-1353-5

6 Mirra SS, Heyman A, McKeel D, Sumi SM, Crain BJ, Brownlee LM, Vogel FS, Hughes JP, van Belle G, Berg L (1991) The Consortium to Establish a Registry for Alzheimer's Disease (CERAD). Part II. Standardization of the neuropathologic assessment of Alzheimer's disease. Neurology 41: 479-486 Doi 10.1212/wnl.41.4.479

7 Montine TJ, Phelps CH, Beach TG, Bigio EH, Cairns NJ, Dickson DW, Duyckaerts C, Frosch MP, Masliah E, Mirra SSet al (2012) National Institute on Aging-Alzheimer's Association guidelines for the neuropathologic assessment of Alzheimer's disease: a practical approach. Acta Neuropathol 123: 1-11 Doi 10.1007/s00401-011-0910-3

8 Thal DR, Rub U, Orantes M, Braak H (2002) Phases of A beta-deposition in the human brain and its relevance for the development of AD. Neurology 58: 1791-1800 Doi 10.1212/wnl.58.12.1791
